# One-Step LCVD Fabrication of Binder-Free Porous Graphene@SiC Heterostructures for Lithium-Ion Battery Anodes

**DOI:** 10.3390/ma18184341

**Published:** 2025-09-17

**Authors:** Song Zhang, Feiyang Ji, Wei Huang, Chitengfei Zhang, Chongjie Wang, Cuicui Li, Qingfang Xu, Rong Tu

**Affiliations:** 1 State Key Laboratory of Advanced Technology for Materials Synthesis and Processing, Wuhan University of Technology, Wuhan 430070, China; kobe@whut.edu.cn (S.Z.); 281286@whut.edu.cn (F.J.); huangwei53@whut.edu.cn (W.H.); 211591@whut.edu.cn (C.W.); 278268@whut.edu.cn (C.L.); turong@whut.edu.cn (R.T.); 2 Hubei Longzhong Laboratory, No.101 Luming Road, Xiangyang 441000, China; zctf@foxmail.com; 3 Hubei Technology Innovation Center for Advanced Composites, Wuhan University of Technology, Wuhan 430070, China

**Keywords:** lithium-ion battery, graphene@SiC heterostructure, binder-free anode, laser chemical vapor deposition (LCVD)

## Abstract

The potential of silicon carbide (SiC) as a promising high-capacity and stable anode material is hindered by poor electronic conductivity and slow lithium diffusion kinetics. Here, we report a one-step laser chemical vapor deposition (LCVD) process to directly synthesize porous graphene@SiC heterostructures on carbon fiber substrates. This in situ method yields an integral, binder-free electrode architecture that enhances mechanical robustness against pulverization. A critical feature of this heterostructure is the built-in electric field at the graphene–SiC interface, which is revealed by theoretical calculations to significantly accelerate charge transport and lithium-ion diffusion. The resulting anode delivers a high reversible capacity of 668 mAh·g^−1^ after 100 cycles at 0.1 A·g^−1^. More remarkably, a unique multi-stage activation mechanism is discovered, leading to an unprecedented capacity rebound to 735 mAh·g^−1^ after cycling at rates up to 5 A·g^−1^. This activation process is observed to accelerate with increasing current density in the 0.1–2 A·g^−1^ range. Furthermore, post-cycling analysis via XRD, TEM, and XPS confirms both the structural durability of the electrode and a reversible lithium intercalation mechanism, providing a critical foundation for the future design of high-performance LIB anodes.

## 1. Introduction

The development of high-energy-density LIBs for next-generation storage applications is constrained by the limited theoretical capacity (372 mAh·g^−1^) of conventional graphite anodes [1,2]. This has motivated the exploration of a wide range of alternative anode materials, including Si [3], Sn [4], Ge [5], and various metal oxides such as Fe_2_O_3_ [6] and TiO_2_ [7]. While many of these materials offer significantly higher capacities, their widespread adoption is hindered by the critical challenge of dramatic volume change during lithiation [3,6]. This issue often leads to electrode pulverization, loss of electrical contact, and rapid capacity fade [1,2]. Among these diverse options, silicon carbide (SiC) has emerged as a compelling candidate due to its exceptional structural stability derived from robust covalent bonding characteristics [8,9,10]. SiC possesses a layered crystalline structure wherein each atom is tetrahedrally bonded to four atoms of the opposing element, creating a highly ordered and stable framework. With C–C and Si–Si bond distances of approximately 3.08 Å and a Si–C bond distance of 1.89 Å, this lattice arrangement provides interstitial pathways sufficient for the diffusion of small ions like Li^+^ [11,12,13].

Recent theoretical investigations based on first-principles have strongly corroborated the intrinsic lithium storage potential of SiC. Chen et al. [14] systematically investigated various SiC polytypes, revealing that Li^+^ can readily intercalate and occupy interstitial sites with tetrahedral symmetry. Their work further identified the 3C-SiC (111) surface as the most kinetically favorable for Li-ion diffusion, predicting a capacity of 223.4 mAh·g^−1^ for a two-layer Li adsorption model. Fatima et al. [15] predicted a favorable Li insertion voltage of 1.85 V for layered SiC, corresponding to a high capacity of 699 mAh·g^−1^, and crucially, a much lower volume expansion than that of pure silicon. Furthermore, research on specific nanostructures, such as the SiC nanowires studied by Tang et al., has demonstrated excellent post-lithiation mechanical resilience and a theoretical capacity reaching 668.42 mAh·g^−1^ for the SiC nanowires grown along the [111] direction [16]. Collectively, these computational works affirm the promise of SiC material as a stable, high-capacity anode and provide a robust theoretical foundation for its experimental development.

However, early studies deemed SiC to be electrochemically inert, functioning primarily as a structural stabilizer in composite systems rather than an active Li^+^ host [17,18,19]. Subsequent investigations revealed that this underperformance stems from intrinsic material limitations, notably a high surface lithiation energy barrier, poor electronic conductivity, and sluggish ion diffusion kinetics, which impede its effectiveness when employed as a standalone anode [8,20,21,22]. For instance, anodes fabricated from commercial SiC powders delivered a modest capacity of only 287.6 mAh·g^−1^ after 200 cycles at 100 mA·g^−1^ [8], while amorphous SiC films, despite a promising initial capacity of 917 mAh·g^−1^ at 0.3 C, suffered significant decay to 376 mAh·g^−1^ after 100 cycles with only 41% retention [22]. To overcome these limitations, extensive research has focused on advanced material engineering strategies. Hu et al. [21] designed a SiC@SiO_2_ core–shell nanowire composite with bead-curtain-like morphology, which achieved a stable specific capacity of 754 mAh·g^−1^ with 92.3% retention after 100 cycles at 268 mA·g^−1^. Similarly, Leonova et al. [23] synthesized carbon/SiC composite electrodes that delivered a discharge capacity of 328 mAh·g^−1^ following 100 cycles at C/2 current rate. In another approach, Sun et al. [8] fabricated EG@SiC electrodes through Schottky junction engineering to activate electrochemically inert SiC, achieving a sustained discharge capacity of 681.8 mAh·g^−1^ after 200 cycles at 0.1 A·g^−1^, corresponding to 78.9% capacity retention relative to the second cycle. More recently, Sun et al. [24] demonstrated that tuning the interfacial interaction at an atomic scale, by epitaxially growing N-doped graphene on SiC particles, could unlock an exceptional performance of 1197.5 mAh g^−1^ after 200 cycles at 0.1 A·g^−1^. Although the optimized structural design has enhanced performance, achieving high discharge specific capacity while maintaining stability remains a persistent challenge due to its reliance on complex fabrication processes [8,21,25,26]. Compounding these challenges, the precise lithium storage mechanism within SiC is still debated. Some researchers propose a conversion reaction where SiC first transforms into active silicon, which then undergoes an alloying reaction with lithium for energy storage [22,27], while others suggest a direct intercalation mechanism [8,9,20]. This highlights the need for further fundamental investigations.

The development of SiC–carbon composites offers a viable pathway to overcome the intrinsic limitations of SiC anodes. This approach is particularly well-aligned with our previous research demonstrating laser chemical vapor deposition (LCVD) as a powerful technique for the in situ synthesis of high-performance SiC–graphene heterostructures. Notably, Xu et al. [28] demonstrated in situ fabrication of <111>-oriented graphene/SiC composite films via LCVD, achieving a remarkable electrical conductivity of up to 7.61 × 10^5^ S/m. Cai et al. [29] confirmed the capability of LCVD in creating desirable porous architectures, which is beneficial for electrochemical applications due to enhanced surface area. Indeed, the practical electrochemical advantages of such LCVD-synthesized SiC–graphene materials have been compellingly demonstrated in supercapacitor applications, which showcased excellent rate capability, high capacitance, and robust long-term cycling stability [30,31]. The conductive, hierarchically porous, and electrochemically robust SiC-graphene composites with well-integrated interfaces prepared via LCVD underscore the unique potential as anodes in next-generation lithium-ion batteries. These findings underscore the unique potential of LCVD in fabricating conductive, hierarchically porous, and electrochemically robust SiC–graphene composites with well-integrated interfaces, which provide a strong foundation for investigating their performance limits within advanced lithium-ion battery systems.

Leveraging these established capabilities, this study reports the one-step LCVD fabrication of binder-free, porous graphene@SiC heterostructure films on carbon fiber paper (CP) for their direct application as anodes in lithium-ion batteries. We conduct a comprehensive investigation of the physicochemical properties of the material and thoroughly evaluate its electrochemical performance as a LIB anode, focusing on capacity, stability, rate capability, and significantly, a unique multi-stage activation mechanism observed during cycling. To elucidate the origins of the favorable electrochemical properties exhibited by these composites, this work combines comprehensive experimental characterization with density functional theory (DFT) calculations to probe the interfacial electronic interactions and propose a detailed interpretation of the fundamental lithium storage mechanism in the graphene@SiC anode.

## 2. Experimental Section

### 2.1. Growth of Graphene@SiC Composite Films

Porous graphene@SiC composite films were synthesized on carbon fiber paper (GDS180S, CeTech, Taichung, Taiwan, 1 cm × 2 cm, 0.19 mm) through LCVD. First, the carbon fiber paper substrate was ultrasonically cleaned in acetone and ethanol (both analytical reagents, >99.7%) for 10 min each at 298 K, followed by drying at the same temperature for 1 h to remove surface impurities. The pretreated substrate was subsequently placed in a cold-wall reactor. Hexamethyldisilane (HMDS, or Si_2_C_6_H_18_), with a stoichiometric Si:C ratio of 1:3, served as the sole precursor. The carbon-rich decomposition of HMDS was the primary reason for the formation of the graphene structure. The continuous-wave laser, maintained at a power of approximately 14.0 W, generated synergistic thermal and photochemical effects. Under the thermal effect of the laser, the higher saturated vapor pressure of Si at the deposition temperature caused it to preferentially sublimate, enriching the surface with carbon. Additionally, the synergistic action of laser photo-etching and hydrogen etching (3000 sccm) at the boundaries of the SiC nanostructures created Si vacancies, which facilitated the epitaxial growth of C-C bonds to form the graphene structure [28,30,32]. A controlled thermal ramp (~573 K/min) was applied to maintain the deposition process at 1423 K and 1400 Pa for 4 min. After deposition, the sample was cooled to 373 K under hydrogen flow before retrieval. The active material loading was determined gravimetrically by measuring the mass difference in the substrate before and after synthesis, with an average value of approximately 0.8 mg·cm^−2^.

### 2.2. Characterizations and Electrochemical Measurements

A comprehensive characterization strategy was utilized to evaluate the graphene@SiC composites pre- and post-cycling in LIBs. Crystallographic and compositional evaluations employed X-ray diffraction (XRD, Rigaku Ultima III, Rigaku, Tokyo, Japan) and Raman spectroscopy (Horiba LabRAM HR Evolution system with a 532 nm laser, Horiba Scientific, Palaiseau, France), while X-ray photoelectron spectroscopy (XPS, AXIS SUPRA+, Kratos Analytical, Manchester, UK) interrogated surface chemistry and bonding configurations. Morphological features, including cross-sectional and surface topography, were resolved via scanning electron microscopy (SEM, Quanta-250, Hillsboro, Oregon, USA), with transmission electron microscopy (TEM, JEM-2100UHR, JEM-2100UHR, JEOL, Tokyo, Japan) providing atomic-scale structural insights. The acquired data were processed using Origin 2025b software.

To determine the electrical conductivity of the prepared films, an RTS-9 four-probe tester (Guangzhou 4-Probe Tech. Co., Ltd., Guangzhou, China) was used to measure the sheet resistance and conductivity. For electrochemical testing, the prepared graphene@SiC on CP composite electrodes were directly sectioned into 1 × 1 cm^2^ specimens, without the use of traditional binders or conductive agents. A 1 M LiPF_6_ solution in ethylene carbonate/dimethyl carbonate (EC/DMC, 1:1 *v*/*v*) served as the electrolyte. All battery components were assembled in an argon-filled glove box (H_2_O, O_2_ < 1 ppm). Electrochemical measurements were conducted using a NEWARE CT-4008Tn battery testing system with galvanostatic charge/discharge cycling conducted at current densities of 100 mA·g and 1 A·g within the voltage window of 0–3.0 V (vs. Li/Li^+^) at 298 K. Cyclic voltammetry (CV) analysis was simultaneously implemented using a scan rate of 0.1 mV/s across the same potential range at 298 K.

Computational analyses (Gengzi Supercomputing Platform) were conducted using the Vienna Ab initio Simulation Package (VASP, University of Vienna, Vienna, Austria), employing DFT with Kohn–Sham formalism and pseudopotentials [33,34]. The calculations are based on idealized, defect-free crystalline structures at a theoretical temperature of 0 K, and do not explicitly include thermal effects or the complex electrode–electrolyte interface present in a working battery [14]. Furthermore, the lithium interaction was modeled at the dilute limit (a single Li atom) to probe the fundamental adsorption behavior on a simplified 2D surface. Core-electron and valence interactions were modeled via the Projector Augmented Wave (PAW) approach [35], while exchange-correlation effects were approximated using the generalized gradient approximation (GGA) with the Perdew–Burke–Ernzerhof (PBE) functional [36]. A plane-wave energy cutoff of 560 eV was enforced, alongside Γ-centered k-meshes (7 × 7 × 1) for Brillouin zone integration. Structural optimizations utilized a 4 × 4 supercell geometry, with convergence thresholds defined at 0.01 eV/Å for atomic forces and 1 × 10^−5^ eV for total energy. To mitigate periodic boundary artifacts, a vacuum spacing exceeding 15 Å was implemented [37]. For density of states (DOS) computations, a refined k-point grid (13 × 13 × 1) and tetrahedron integration were applied. Dispersion forces were accounted for using the DFT-D3 correction method [37,38], and data postprocessing utilized VASPKIT (Institute of Physics, Chinese Academy of Sciences, Beijing, China) and VESTA (National Institute for Materials Science, Tsukuba, Japan) tools for visualization and analysis [39,40].

## 3. Result and Discussion

**Figure 1:**
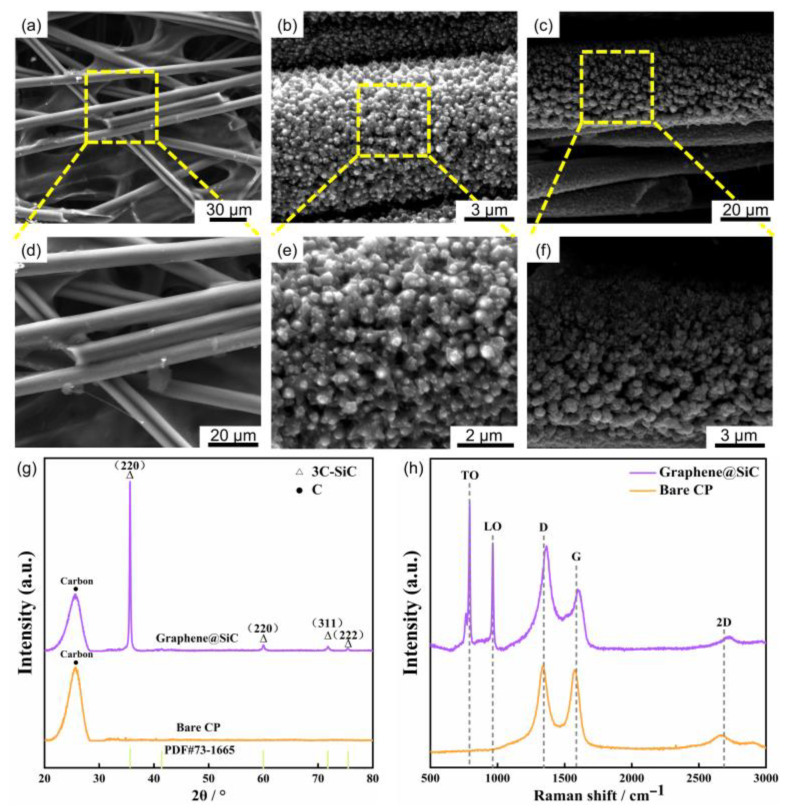
Structural characterization of graphene@SiC porous films: (**a**) FESEM image of bare carbon paper (CP); (**b**) surface and (**c**) cross-sectional views of the composite film. (**d**–**f**) Higher-magnification images of the regions highlighted by the yellow boxes in (**a**–**c**). (**g**) Comparative XRD patterns and (**h**) Raman spectra between as-deposited film and bare CP.

The structural morphology of the as-synthesized film was characterized by using field-emission scanning electron microscopy (FESEM), as presented in Figure 1. The pristine CP substrate consisted of an interconnected network of carbon microfibers (Figure 1a,d), providing a porous scaffold for subsequent material deposition [21,41]. After the LCVD process, this substrate became uniformly coated with a hierarchical nanostructure exhibiting a flower-like nanoforest structure (Figure 1b,e). The porous structure provided dual benefits by expanding the electrode–electrolyte interfacial area with abundant active sites to accelerate reaction kinetics, while also offering mechanical compliance to accommodate multidirectional volume variations during Li^+^ insertion and extraction [42,43]. Furthermore, cross-sectional FESEM analysis revealed a tapered morphology, with broader bases gradually narrowing toward the top (Figure 1c,f), suggesting a Volmer–Weber growth mechanism [44,45].

The chemical composition of this film was confirmed by X-ray diffraction (XRD) and Raman spectroscopy. The XRD pattern in Figure 1g displayed distinct diffraction peaks at 35.7°, 59.9°, 71.8°, and 75.5°, which correspond to the (111), (220), (311), and (222) planes of crystalline 3C-SiC (JCPDS 73-1665), respectively [32,46]. Using Bragg’s Law, the d-spacing calculated from the most intense peak at 35.7° was determined to be 2.5 Å, which corresponds to the 3C-SiC(111) plane. The prominence of the (111) peak compared to other planes indicates a strong <111> preferred growth orientation, consistent with the lowest surface energy configuration in face-centered cubic (FCC) crystal systems [46]. Furthermore, the average crystallite size of SiC was calculated to be approximately 40 nm using the Scherrer equation based on the full width at half maximum (FWHM) of the (111) peak obtained through multi-peak fitting with Origin software. Complementary analysis by Raman spectroscopy (Figure 1h) further corroborated the XRD results, displaying characteristic Si-C bond peaks for 3C-SiC (792 cm^−1^ TO and 1090 cm^−1^ LO) alongside the D (1361 cm^−1^), G (1602 cm^−1^), and 2D (2782 cm^−1^) bands of graphene [29,46,47,48]. Notably, these graphene-related bands showed a distinct blue shift compared to their positions on the bare CP substrate (1350 cm^−1^, 1597 cm^−1^, and 2690 cm^−1^, respectively) [49]. This effect may be attributed to structural strain induced during the deposition process, or to the formation of graphene co-deposited with SiC [49].

**Figure 2:**
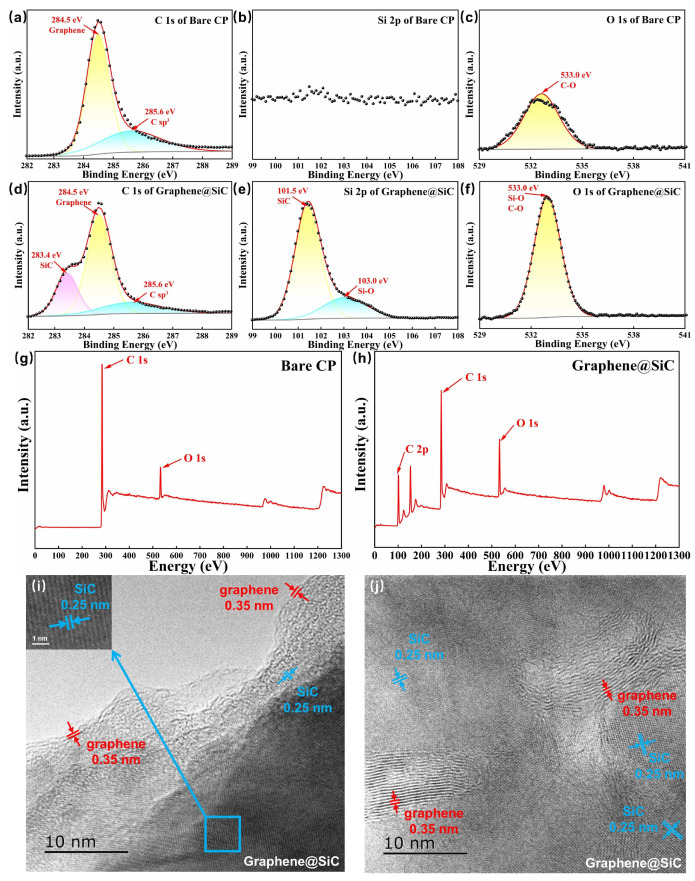
(**a**–**c**) High-resolution XPS spectra of bare CP: (**a**) C 1 s, (**b**) Si 2p, and (**c**) O 1 s; (**d**–**f**) XPS spectra of graphene@SiC: (**d**) C 1 s, (**e**) Si 2p, and (**f**) O 1 s; (**g**,**h**) survey spectra of bare CP (**g**) and graphene@SiC (**h**); (**i**,**j**) High-resolution TEM micrographs of graphene@SiC composite.

To investigate the surface chemistry and microstructure, X-ray photoelectron spectroscopy (XPS) and transmission electron microscopy (TEM) were performed (Figure 2). For the bare CP substrate, the C 1s spectrum showed a strong peak at 284.5 eV, attributed to sp^2^-hybridized C-C bonds, and a smaller component at 285.6 eV, likely from sp^3^-hybridized defects [8,24]. These features indicated the highly graphitized nature of the carbon paper. In contrast, the spectrum for the deposited films retained these characteristic peaks while exhibiting an additional distinct peak at 283.4 eV, attributed to carbon atoms in the SiC environment, confirming the co-deposition of carbon and SiC [8,24]. The Si 2p spectrum presented a characteristic Si-C peak at 101.5 eV, alongside a Si-O peak at 103.0 eV which indicated surface oxidation from residual oxygen (~10^−3^ Pa) during the LCVD process. In addition, the O 1 s spectrum at 533.0 eV exhibited a sharper profile and increased intensity compared to the bare CP, indicating a higher surface oxygen content due to the enhanced activity of the graphene and SiC [50]. An examination of the C 1 s fine spectrum revealed that the FWHM of the sp^2^-hybridized C-C peak was 1.5 eV, significantly narrower than that of the bare CP (1.8 eV), suggesting that the ordered epitaxial growth of carbon effectively suppressed disordered carbon accumulation on the substrate surface. Complementing this chemical analysis, high-resolution TEM imaging revealed the physical arrangement of these components at the nanoscale (Figure 2i,j). A layer spacing of 0.25 nm corresponds to the (111) plane of 3C-SiC, while a 0.35 nm spacing corresponds to the graphene structure [8]. It was also observed that graphene existed not only on the SiC surface but was also distributed internally. This tight integration, a direct result of the one-step LCVD synthesis, ensured robust electrical contact and was expected to enhance interfacial charge kinetics, which is critical for electrochemical performance.

**Figure 3:**
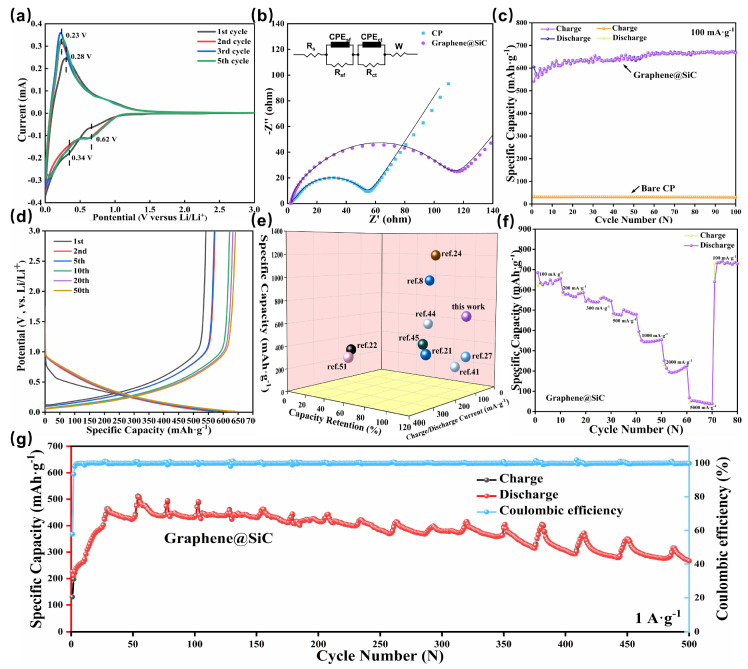
Electrochemical characterization of graphene@SiC composite anode: (**a**) Cyclic voltammograms at 0.1 mV/s. (**b**) EIS curves with equivalent circuits for batteries containing bare CP and graphene@SiC electrodes. (**c**) Cycling performance at 100 mA/g. (**d**) Galvanostatic charge–discharge profiles for the selected cycles at 0.1 A/g. (**e**) Comparative capacity retention with reported SiC-based anodes. (**f**) Rate capability testing. (**g**) Long-term cycling stability at 1 A/g.

To understand the electrochemical behavior of the graphene@SiC anode, cyclic voltammetry (CV) was conducted at a scan rate of 0.1 mV·s^−1^ (Figure 3a). During the initial cathodic scan, a distinct and irreversible peak was observed at approximately 0.34 V, which was attributed to the formation of an inactive SEI layer, a phenomenon consistent with previous reports on SiC-based anodes [9,51]. Although graphene enhances the electrochemical performance of SiC, it does not alter the fundamental Li^+^ storage mechanism. In subsequent cycles, the 0.34 V peak disappeared while a stable lithiation peak developed at approximately 0.62 V, indicating a highly reversible electrochemical reaction. These CV profiles differ significantly from those of pure silicon or carbon anodes [2,3]. Specifically, the stable lithiation peak at 0.62 V is notably higher than the typical alloying peaks of pure Si anodes (~0.1 V) and the delithiation oxidation peaks of pure C anodes (~0.2–0.3 V) [1,2,3]. This suggests that lithiation in our material does not involve Li_x_Si or Li_x_C formation [8,24]. Instead, the structural integrity of the graphene@SiC is likely preserved due to the formation of stable, potential-dependent phases during cycling, consistent with previous studies on SiC anodes [8,24].

Electrochemical impedance spectroscopy (EIS) was employed to analyze the interfacial dynamics of the graphene@SiC anode (Figure 3b) using an equivalent circuit model (top-left inset), where R_s_ represents the ohmic resistance, R_sf_ and R_ct_ denote the SEI film resistance and charge transfer resistance, respectively, and W (Warburg impedance) reflects lithium-ion diffusion behavior [43,52]. The fitting results reveal a decrease in R_s_ from 2.81 to 2.26 Ω, confirming the enhanced conductivity facilitated by the interconnected graphene network. In contrast, the R_sf_ exhibits a significant increase from 1.83 to 11.53 Ω, indicating a higher ionic transport resistance resulting from the formation of a denser SEI layer on the high-surface-area surface of the composite [43,53]. Meanwhile, the R_ct_ increases from 48.15 to 92.05 Ω, primarily attributed to the inherent semiconductor nature of SiC and the additional interfacial resistance at the graphene-SiC junction. Remarkably, the R_ct_ of the composite films still outperforms those of conventional high-capacity anodes despite these compromised kinetic limitations [43,53].

The electrochemical cycling performance of the graphene@SiC composite anode was evaluated at a current density of 100 mA·g^−1^ (Figure 3c). In the first cycle, the anode delivered an initial discharge capacity of 604.72 mAh·g^−1^ and a charge capacity of 541.77 mAh·g^−1^. The observed 10.4% irreversible capacity loss was primarily attributed to SEI formation, as evidenced by the characteristic high-voltage slope in the initial charge–discharge profile (Figure 3d) [54]. Subsequently, the coulombic efficiency stabilized above 99%, and consistent voltage plateaus appeared, indicating stable polarization control. Notably, the reversible capacity exhibited a progressive increase to approximately 650 mAh·g^−1^ after 55 cycles and 668 mAh·g^−1^ after 100 cycles, exceeding that of the bare CP anode by over thirtyfold. This remarkable enhancement could be attributed to the gradual activation mechanism of the SiC component. The unique capacity growth pattern observed in this study aligns with previous reports on SiC-based anodes [9,20,24]. As illustrated in Figure 3e, the graphene@SiC anode developed in this work demonstrated exceptional reversible specific capacity over 100 cycles, along with superior capacity retention compared to previously reported SiC-based systems, particularly those with similar or higher initial capacities. This performance advantage became more pronounced when compared with experimental results exhibiting comparable or greater reversible capacities, highlighting the exceptional electrochemical stability of this material.

The rate capability of the graphene@SiC anode was evaluated by cycling the cell at progressively increasing current densities from 100 mA·g^−1^ to 5000 mA·g^−1^, as shown in Figure 3f. At an initial current density of 100 mA/g, the anode delivered a discharge capacity of 685.24 mAh·g^−1^ and a charge capacity of 622.71 mAh·g^−1^, with a progressive capacity activation consistent with the trend observed in previous cycling tests. As the current density increased, the capacity showed a stepwise decline to 580 mAh·g^−1^ at 200 mA·g^−1^, 550 mAh·g^−1^ at 300 mA·g^−1^, 480 mAh·g^−1^ at 500 mA·g^−1^, and dropped to 347 mAh·g^−1^ at 1000 mA·g^−1^. Intriguingly, even under a high rate of 2000 mA/g, after an initial decrease, the capacity recovered from 195.55 mAh·g^−1^ to 225 mAh·g^−1^, suggesting enhanced SiC activation under high-rate cycling that improves Li^+^ diffusion kinetics and interfacial reactivity [20,21]. The capacity fell to 48 mAh·g^−1^ at 5000 mA·g^−1^, indicating that structural optimization needs for high-rate compatibility. The rate-dependent fading revealed that while the porous framework enhances ionic kinetics via shortened Li^+^ paths at low currents, it cannot sustain rapid ion insertion/extraction at higher rates. Concurrently, mechanical stress induced by high-rate cycling may compromise the graphene–SiC interfacial bonding, increasing charge transfer resistance. Remarkably, upon returning to 100 mA·g^−1^, the capacity rebounded to 735 mAh·g^−1^ with 7.3% enhancement over the initial value, demonstrating that the capacity loss under high rates stems from kinetic limitations rather than structural degradation. The exceptional reversibility observed here, which contrasts markedly with conventional Si-based anodes, suggests a self-adaptive interface mechanism, providing critical guidance for developing stable, high-power anode architectures.

The cycling performance of the graphene@SiC anode was tested under a stringent current density of 1 A·g^−1^, with results presented in Figure 3g. The initial discharge capacity was notably low at 226 mAh·g^−1^, with a charge capacity of 130 mAh·g^−1^, indicating limited initial activation under high-rate conditions, likely due to reduced electron transfer efficiency caused by the elevated current rate [2]. However, with increasing cycle numbers, the discharge capacity rose rapidly, peaking at 507 mAh·g^−1^, mirroring the activation trend observed at lower current densities and suggesting a consistent activation mechanism for SiC across different rates. The accelerated capacity increase at this high rate may stem from enhanced surface reaction kinetics, swiftly unlocking the active sites of SiC, though this could also induce partial structural damage. Post-peak, the capacity exhibited distinct fluctuations: the charge–discharge capacity gradually declined, followed by abrupt increases, repeating this pattern multiple times. We hypothesize that this unique behavior may arise from stepwise activation of SiC, where inner active material is progressively exposed through surface corrosion or delamination [9,20]. This is also possibly influenced by significant volume changes inherent to silicon-based materials, which can induce periodic structural strain and impact capacity stability [3]. After 500 cycles at 1 A·g^−1^, the capacity stabilized at approximately 380 mAh·g^−1^, retaining 273 mAh·g^−1^ by the 500th cycle, reflecting reasonable long-term stability despite fluctuations. The initial rapid capacity rise and subsequent oscillations suggest a dynamic interplay of activation and degradation processes, potentially exacerbated by SEI film instability under high-rate conditions, offering insights into the resilience and limitations of this material at elevated current densities.

**Figure 4:**
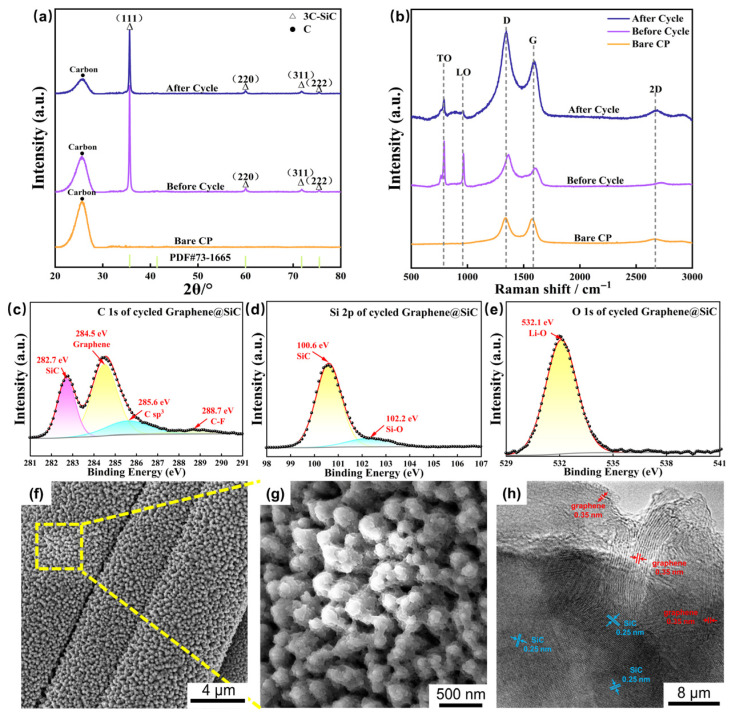
(**a**) XRD patterns of graphene@SiC composite electrodes (before/after cycling) and bare CP; (**b**) Raman spectra of graphene@SiC composite electrodes (before/after cycling) and bare CP; (**c**) XPS C 1s spectrum of cycled graphene@SiC; (**d**) XPS Si 2p spectrum of cycled graphene@SiC; (**e**) XPS O 1s spectrum of cycled graphene@SiC; (**f**) surface SEM image of cycled electrode; (**g**) magnified SEM image; (**h**) TEM image of cycled electrode.

Post-mortem analysis via XRD and Raman spectroscopy elucidated the structural evolution of the graphene@SiC electrode after 100 cycles at 100 mA·g^−1^ (Figure 4a,b). The 3C-SiC component demonstrated exceptional stability, with XRD showing only a minor reduction in diffraction intensity and a negligible FWHM increase from 0.209° to 0.210°. Raman analysis corroborated this, revealing stable SiC modes alongside significant alterations in the graphene component. Specifically, the D, G, and 2D bands of graphene showed a redshift to 1350, 1597, and 2690 cm^−1^, and the I_D_/I_G_ ratio increased from 1.76 to 1.92, indicating lattice strain relaxation and increased disorder [55]. The sharp narrowing of the 2D peak FWHM suggested altered interlayer coupling, likely due to lithium intercalation [56,57].

The chemical and microstructural state of the graphene@SiC electrode after cycling was further investigated by a combination of XPS, SEM, and TEM (Figure 4). XPS showed a Si-C peak shift in Si 2p (100.6 eV, Δ = −0.9 eV) and C 1s (282.7 eV, Δ = −0.7 eV), evidencing Li_x_SiC formation and strain relaxation, while C-F emergence (288.7 eV) and O 1 s shift (532.1 eV) confirmed SEI components (e.g., Li_2_CO_3_). SEM (Figure 3f,g) depicted a retained flower-like nanoforest morphology, albeit with flattened crystalline petals due to cycling-induced densification. TEM revealed intact graphene coatings on SiC particles, with unchanged lattice fringes (0.25 nm for SiC(111), 0.35 nm for graphene), demonstrating remarkable nanoscale stability post-cycling. This synergy demonstrated that SiC enabled high-capacity lithium storage with minimal degradation, complemented by the capacity of graphene to preserve interfacial integrity and alleviate expansion stresses, presenting superior performance over conventional silicon anodes susceptible to pulverization [58].

**Figure 5:**
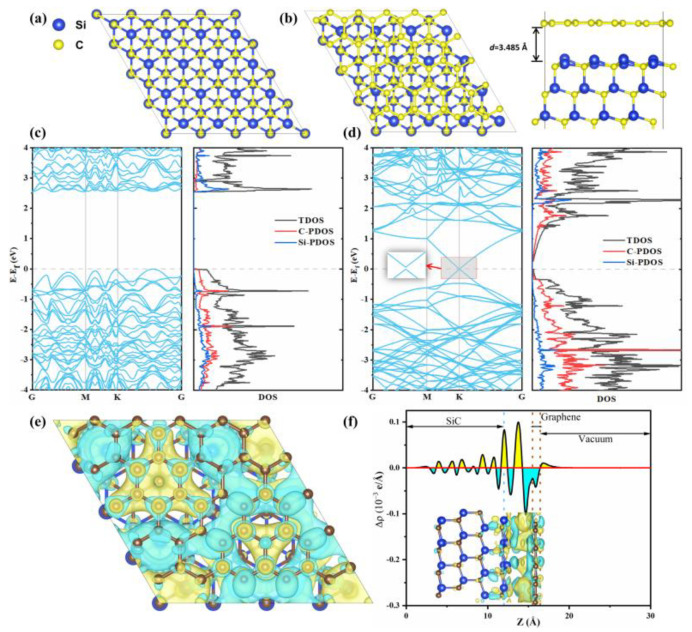
(**a**) Top views of SiC. (**b**) Top and side views of graphene@SiC. (**c**,**d**) Band structure (left) and PDOS (right) of SiC and graphene@SiC, respectively. The inset in (**d**) shows a magnified view of the band structure near the Dirac point. (**e**) Charge density difference (Δρ) with an isosurface level of 0.001 |e|/Å^3^ (yellow: charge accumulation; blue: charge depletion). (**f**) Plane-averaged electron density difference along the z-axis of graphene@SiC.

To investigate the interfacial charge behavior in the graphene@SiC heterostructure, DFT calculations were conducted on optimized models of pristine SiC, graphene, and the graphene@SiC structure. SiC adopts a tetrahedral structure with sp^3^ hybridization (Figure 5a), while graphene features a 2D hexagonal honeycomb lattice. We constructed a graphene@SiC heterostructure by combining a 5 × 5 graphene supercell and a 4 × 4 SiC supercell, resulting in a minimal lattice mismatch of 0.9%. The optimized lattice constants were a = b = 12.344 Å, with an interlayer distance of d = 3.485 Å, in agreement with previous studies [59,60]. Electronic conductivity plays a crucial role in determining the cycling stability and rate performance of electrode materials in LIBs. To explore this, the band structures and partial density of states (PDOS) of SiC and the graphene@SiC heterostructure were analyzed. The band structure revealed that SiC is a semiconductor with an indirect band gap of 2.56 eV (Figure 5c). In the graphene@SiC heterostructure, the Dirac point of graphene at the K point was disrupted, resulting in a small bandgap of approximately 3.2 meV (calculated using GGA-PBE) (Figure 5d).

Charge density difference calculations (Figure 5e,f) were employed to understand the nature of the interlayer interactions. When graphene interacts with SiC, electron transfer occurs, with electrons shifting from graphene toward SiC. This results in an accumulation of negative charge on the SiC side and positive charge on the graphene, generating an intrinsic electric field directed from graphene to SiC [8]. This built-in electric field is highly beneficial, as it concurrently enhances electronic conductivity by facilitating charge transfer, drives Li^+^ diffusion toward SiC to improve insertion/extraction kinetics, and strengthens interfacial bonding for greater structural stability during cycling [61]. Therefore, incorporating graphene significantly enhances the electronic conductivity, making the graphene@SiC composite a promising anode for stable, high-rate LIBs.

**Figure 6:**
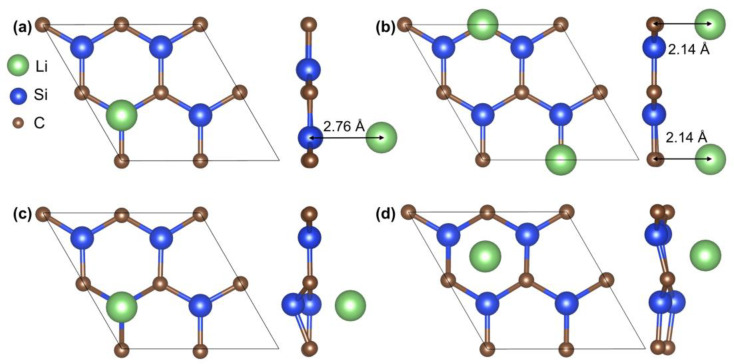
The top view and side view of the SiC sheet with lithium placed at four different adsorption sites: (**a**) atop a silicon atom, (**b**) atop a carbon atom, (**c**) at the bridge site above the Si-C bond, and (**d**) at the hexagonal hollow site.

To elucidate the lithium storage mechanism, the interaction of lithium with a simplified 2D-SiC surface was investigated using a 2 × 2 supercell model. Four potential lithium adsorption sites were systematically analyzed (Figure 6): atop a silicon atom (T1), atop a carbon atom (T2), the bridge site above the Si-C bond (B), and at the hexagonal hollow site (C). Thermodynamic analysis based on electronic energies identified the C-site configuration (−57.34 eV) as the most stable, with all lithiated systems exhibiting enhanced thermodynamic stability relative to pristine SiC (−56.22 eV). Mechanically (Table 1), all configurations satisfied the Born stability criteria (C11 > 0 and C11 > |C12|) post-lithiation, confirming their structural stability. However, lithium adsorption generally reduces stiffness, with T2 exhibiting the most significant decline, attributed to strong Li-C interactions causing lattice distortion. T1 retained properties closest to pristine SiC, while B and C showed moderate reductions. This mechanical degradation correlates with the experimentally observed stepwise capacity release, particularly prominent at high current densities, where rapid Li^+^ intercalation leads to localized lattice stress and periodic microcrack formation-passivation cycles. Based on these findings, an intercalation mechanism is proposed, as depicted by the following reaction: graphene@SiC + xLi^+^ + xe^−^ → graphene@Li_x_SiC [8,22]. This mechanism is supported by the absence of characteristic Si or C lithiation peaks in CV profiles, with SEM, XPS, and TEM analyses confirming the microstructural stability of the graphene@SiC composite before and after cycling.

Looking ahead, future research should focus on optimizing the graphene–SiC interface to further enhance electron transport and mechanical robustness. Exploring alternative electrolyte systems that accommodate volume changes during SiC lithiation and investigating the long-term cycling stability of graphene@SiC composites under diverse operating conditions are critical. Notably, the observed multi-stage activation mechanism opens avenues for innovative battery technologies, such as dynamic recharging protocols or adaptive energy management systems, where progressive capacity unlocking could enable self-repairing electrodes or rapid power replenishment. These advancements, coupled with scalable LCVD fabrication, will accelerate the realization of high-performance SiC-based anodes, propelling next-generation lithium-ion batteries toward unprecedented energy density and operational resilience.

## 4. Conclusions

This study demonstrates the exceptional potential of binder-free graphene@SiC heterostructured composite anodes, fabricated via a rapid and scalable LCVD process, for high-performance lithium-ion batteries. The optimized anode delivered a remarkable reversible capacity of 668 mAh·g^−1^ after 100 cycles at 0.1 A·g^−1^ with a coulombic efficiency that stabilized above 99%. More importantly, the composite demonstrated superior rate capability and unprecedented cycling stability, retaining an average capacity of 380 mAh·g^−1^ over 500 cycles at an elevated current density of 1 A·g^−1^. A particularly noteworthy observation is the capacity rebound to 735 mAh·g^−1^ at 0.1 A·g^−1^ following high-rate cycling up to 5 A·g^−1^, surpassing its initial performance. This enhancement is attributed to the progressive activation of lithium storage sites in the SiC matrix, enabled by the unique graphene heterostructure that provides both dynamic strain accommodation and intrinsic structural self-repair functionality. The underlying lithium storage mechanism was identified as an intercalation process forming graphene@Li_x_SiC, a conclusion supported by the absence of Si/C lithiation peaks in CV profiles and validated by post-cycling SEM, XPS, and TEM analyses that confirm microstructural integrity. The discovery of this multi-stage activation mechanism not only clarifies the unconventional lithium storage dynamics but also suggests novel operational strategies for next-generation batteries. This behavior could be harnessed to engineer self-optimizing energy storage systems with built-in conditioning capabilities, paving the way for batteries with enhanced energy density and operational lifespan.

## Figures and Tables

**Table 1:** Elastic constants when Li is placed at T1 T2 B and C configurations.

	Elastic Constant (N/m)		Young’s Modulus (N/m)	Shear Modulus (N/m)	Poisson’s Ratio	Electronic Energy (eV)
	C11	C12				
T1	172.25	53.02	155.93	59.62	0.308	−56.78
T2	117.11	96.15	38.17	10.48	0.821	−57.05
B	152.12	38.91	142.16	56.6	0.256	−57.28
C	129.47	42.35	115.62	43.56	0.327	−57.34
Pristine SiC	181.52	55.61	164.48	62.96	0.306	−56.22

## Data Availability

The original contributions presented in this study are included in the article. Further inquiries can be directed at the corresponding author.
